# Novel topical esmolol hydrochloride improves wound healing in diabetes by inhibiting aldose reductase, generation of advanced glycation end products, and facilitating the migration of fibroblasts

**DOI:** 10.3389/fendo.2022.926129

**Published:** 2022-08-23

**Authors:** Sudhir A. Kulkarni, Supreet K. Deshpande, Ashu Rastogi

**Affiliations:** ^1^ Department of Molecular Diabetes, NovaLead Pharma Pvt. Ltd., Pune, India; ^2^ Department of Endocrinology, Post Graduate Institute of Medical Education and research (PGIMER), Chandigarh, India

**Keywords:** diabetes, diabetic foot ulcers, esmolol hydrochloride, wound healing, aldose reductase (ALR), sorbitol, pharmacokinetic

## Abstract

**Aims/Objectives:**

Wound healing in people with diabetes is delayed secondary to impaired nitric oxide generation, advanced glycation end products (AGE), and poor migration of epithelial cells. We developed a novel topical esmolol hydrochloride (Galnobax) and assessed its efficacy for wound healing in streptozocin-induced diabetic hairless rat.

**Methods:**

All experiments were performed at an animal laboratory and tertiary-care research facility. *Ex vivo* aldose reductase inhibition was assessed from enzymes obtained from a bacterial culture (spectrophotometer), sorbitol content in homogenized red blood cells, and AGE in glucose and bovine serum by fluorometry following the addition of esmolol in varying concentrations. A scratch assay of human fibroblasts, endothelial cells, and keratinocytes was assessed under a high-glucose environment and after esmolol by phase-contrast microscopy. The efficacy evaluation of the topical application of Galnobax (14 and 20%) or vehicle was conducted in streptozotocin-induced diabetic hairless rats, and endogenous nitrite and hydroxyproline from homogenized wound tissue were measured along with pharmacokinetic and dermal toxicity in Hanford miniature swine.

**Results:**

Esmolol inhibited the formation of sorbitol by 59% in erythrocytes in comparison to glucose-induced sorbitol levels. AGE generation in bovine serum albumin was reduced at 1 mM esmolol concentrations (2.6 ± 1.7) compared with control (*p* < 0.05) and similar to that of diclofenac (2.5 ± 1.3). Esmolol at 1 and 10 µM enhanced the migration of fibroblasts, epithelial cells, and keratinocytes compared with control. The nitric oxide levels (day 7) were 44 and 112% higher with Galnobax (14%) than those of the diabetic group (*p* < 0.05) and the vehicle control group (*p* < 0.05), respectively. The days 7 and 14 hydroxyproline in the wound was higher by 22 and 44% following Galnobax (14%) compared with the diabetic and vehicle control groups. The wound area exhibited better reduction with Galnobax at 14% up to day 10 follow-up compared with the controls. The pharmacokinetic and dermal toxicity in miniature swine suggested no significant adverse event with Galnobax.

**Conclusions:**

Topical esmolol hydrochloride is a novel, safe, and effective treatment modality that acts through pleotropic mechanisms to hasten wound healing in diabetes.

## Introduction

There are three phases of normal wound healing—namely, inflammation, proliferation, and remodeling. Several processes involved in these phases are impaired in people with diabetes, leading to the chronicity of wound, particularly foot ulcers (DFU) (1). The diabetic wounds exhibit a prolonged inflammatory response from cytokines like IL-6, IL-1β, and TNF-α, which contributes to the abnormal response from cells involved in wound healing like macrophages, endothelial cells, fibroblasts, and keratinocytes (2). In the proliferative phase, a decreased production of growth factors affects the recruitment of keratinocytes, fibroblast, immune cells, and vascular cells which are implicated in angiogenesis and extracellular matrix (ECM) formation. In the remodeling phase, the migration of keratinocytes and crosslinking of collagen get hampered (2). Nitric oxide (NO) generation, which has a vital role in microvascular homeostasis, angiogenesis, epidermal migration, formation of granulation tissue, and collagen deposition in the wound, is also impaired in diabetes (3).

Some existing drugs have been investigated in drug repurposing efforts, such as statins, phenytoin, metformin, propranolol, valsartan, as well as DPP-4 inhibitors, but have limited or no efficacy for wound healing (4–9). Topical phenytoin was shown to reduce the time for granulation tissue in ulcers as well as promote a reduction in the size of ulcers (4). Topical propranolol was shown to promote angiogenesis, induce keratinocyte migration for wound re-epithelialization, and form ECM (5). Topical mevastatin stimulated epithelialization and angiogenesis in animal models by exhibiting the modulation of GR ligands and c-Myc inhibition (6). Topically applied metformin applied to diabetic rats suppresses NF-κB p65 activity and diminishes the expression of MMP2 and MMP9 to accelerate wound healing (7). In diabetic mice, DPP-4 inhibitors have been shown to promote the migration of keratinocytes (8). Treatment with a nano-formulation of valsartan showed enhanced healing in diabetic rats through the COX-2, NF-κB, nitric oxide, TGF-β, and VEGF pathways (9). The complex multifactorial pathways involved in diabetic wound healing with limited pharmacological therapies suggest the need for the development of newer treatment modalities.

Hence, another look at the pathophysiology of diabetic microvascular and macrovascular complications and therapies that could address the overstimulation of the protein kinase C signaling pathway, overproduction and accumulation of advanced glycation end products (AGEs), increased flux through the hexosamine pathway, and increased accumulation of sorbitol, is needed (10). Particularly, molecules targeting the activation of the polyol pathway resulting from hyperglycemia that leads to sorbitol accumulation and the formation of AGEs which contribute to diabetic neuropathy and diabetic wounds require further experiments.

Esmolol hydrochloride is an intravenously administered beta-1-selective (cardioselective) adrenergic receptor blocking agent with a short half-life approved for the treatment of supraventricular tachycardia. We performed extensive computational protein–drug docking experiments with esmolol using GRIP docking method and crystal structure 1EL3 obtained from a protein bank (www.rscb.org). We found that esmolol displayed favorable docking outcomes for the inhibition of aldose reductase (AR), a key enzyme in the pathogenesis of several diabetic complications. Encouraged by the results of the docking studies, we plan to study the effect of esmolol on these pathophysiological targets as well as the key cell types of human fibroblasts, endothelial cells, and keratinocytes involved in wound healing. The present study reports the preclinical development and effect of a novel topical formulation of esmolol hydrochloride (Galnobax) for the treatment of DFU.

## Methods

The animal study protocol was approved by the animal research institute committee (IACUC of Bio-Quant Inc., approval number A/102/Q), and all experiments described in the present study were conducted in accordance with the ethical standards of animal care. The toxicity study in miniature swine was conducted under Good Laboratory Practices (GLP) at Sinclair Research Center, LLC. The skin irritation and skin sensitization studies were carried out at Consumer Product Testing Company Inc. under GLP conditions.

### Docking studies

A docking study of esmolol was performed using a Grid-based PLP docking (GRIP docking) method, as provided in VlifeMDS software (VLifeMDS version 4.1: www.vlifesciences.com) using 3-D crystal structure 1EL3 obtained from a protein data bank (www.rcsb.org). GRIP docking allows the use of existing knowledge by providing ligand-guided as well as cavity-guided options. Using the information regarding receptor cavity and ligand position (if known), it generates a grid around the reference ligand in the active site or the whole active site itself. GRIP docking consists of a pre-computation of grids, sampling a set of initial poses, and search of best possible poses by maximizing the favorable interaction and minimizing the steric unfavorable and repulsive interaction. The 1EL3 structure has a co-crystallized ligand in the active site of the protein which was used as a reference ligand in GRIP docking. In GRIP docking, conformers of esmolol were generated and placed on the grid points of a receptor cavity, and the interaction score of each conformer at grid points was evaluated. The docking scores were reported for best 100 poses in the cavity.

### *Ex vivo* study protocol

#### Aldose reductase inhibition

For the enzyme inhibition studies, purified human recombinant aldose reductase (expressed in *Escherichia coli*) was used for testing the AR inhibitory activity of esmolol hydrochloride by a spectrophotometric method using glyceraldehyde as substrate (11).

#### Materials

DL-glyceraldehyde, glucose, lithium sulfate, 2-mercaptoethanol, NADPH, dimethyl sulfoxide, TC-199 medium (M-3769), sorbitol, sorbitol dehydrogenase, NAD, and glutathione reductase were purchased from Sigma Chemical Company (St. Louis, MO, USA).

##### Purification of recombinant human aldose reductase

Recombinant human aldose reductase was purified from bacterial cultures. Enzyme from expression cultures was extracted and purified essentially as described previously (12) with the exception that affinity chromatography over AffiGel Blue (Bio-Rad) was used as a final purification step.

##### Aldose reductase assay

AR activity was assayed according to the method described in the literature (13). The assay mixture in 1 ml contained 50 μM potassium phosphate buffer, pH 6.2, 0.4 mM lithium sulfate, 5 μM 2-mercapto ethanol, 10 μM DL-glyceraldehyde, 0.1 μM NADPH, and enzyme preparation (recombinant enzyme). Appropriate blanks were employed for corrections. The assay mixture was incubated at 37°C and initiated by the addition of NADPH at 37°C. The change in absorbance at 340 nm due to NADPH oxidation was followed in a Cary Bio 100 spectrophotometer.

##### Inhibition studies

For the inhibition studies, a concentrated stock of esmolol hydrochloride was prepared in water. Various concentrations of esmolol hydrochloride were added to the assay mixture and incubated for 5–10 min before initiating the reaction by NADPH as described above. The percent of inhibition with a test compound was calculated considering that the AR activity in the absence of an inhibitor was 100%. The concentration of the test sample giving 50% inhibition (IC50) was then estimated.

#### Inhibition of sorbitol formation in erythrocytes

Esmolol hydrochloride was tested for its potential to inhibit the formation of sorbitol in red blood cells (RBC) by the method reported by Malone et al. (14). The method in brief is discussed below.

##### *In vitro* incubation of RBC

Five milliliters of blood sample was obtained from a healthy male volunteer on overnight fasting in heparinized tubes. Red blood cells were separated by centrifugation and washed three times with isotonic saline at 4°C. The washed RBC were suspended in Kreb’s ringer bicarbonate buffer, pH 7.4 (preequilibrated with 5% CO_2_), and incubated at 37°C in the presence of 5% CO_2_ for 3 h under normal (3.0 mM) and high glucose (30 mM) conditions. The effect of esmolol hydrochloride on sorbitol accumulation was evaluated by incubating the RBC with IC_50_ concentrations of esmolol hydrochloride for the inhibition of aldose reductase.

##### Estimation of sorbitol in RBC

At the end of the incubation period, RBC was homogenized in nine volumes of 0.8 M perchloric acid. The homogenate was centrifuged at 5,000 × g at 4°C for 10 min, and the pH of the supernatant was adjusted to 3.5 with 0.5 M potassium carbonate. The sorbitol content of the supernatant was measured by fluorometric method as described previously (14) using a spectrofluorometer (Jasco-FP-6500).

#### Inhibition of advanced glycation end products

Esmolol hydrochloride was tested for its potential to inhibit the formation of AGEs using an *in vitro* fluorometric method (15). A reaction mixture of glucose and bovine serum albumin was made up to a final volume of 6 ml, mixed with or without the drug, and then autoclaved at conditions of 15 lbs pressure for 10 min at 121°C. Fluorescence was measured at the excitation wavelength of 360 nm and the emission wavelength of 440 nm. Afterwards, the samples were cooled to room temperature.

### *In vitro* study protocol

#### Scratch assay of human fibroblasts, endothelial cells, and keratinocytes

High blood glucose levels hamper the migration of human fibroblasts, endothelial cells, and keratinocytes that impair wound healing (16–20). The ability of esmolol hydrochloride to promote the migration of human fibroblasts, endothelial cells, and keratinocytes under a high-glucose environment was evaluated by scratch assay to evaluate the ability of esmolol hydrochloride in promoting the migration of key cell types involved in wound healing, *viz*., fibroblasts, endothelial cells, and keratinocytes. The percent migration of each cell type at various hours was compared with the untreated control and positive control at various concentrations (0.01 to 1,000 μM) of esmolol hydrochloride. The detailed protocol and cytotoxicity results are provided in the **Supplementary File**.

An actively growing culture of HFF-1 (human dermal fibroblasts), Ea.hy926 (human endothelial cells), and HaCaT (human keratinocytes) cell lines in Dulbecco’s modified Eagle’s medium containing 25 mM glucose supplemented with 10% fetal bovine serum was prepared. The cells were counted through a hemocytometer and were seeded at a density of 0.08 × 10^6^ (HFF-1), 0.1 × 10^6^ (Ea.hy926), and 0.2 × 10^6^ (HaCaT Cells) cells/well/ml of culture medium. The plates were incubated overnight followed by serum starvation for 24 h. Scratch wounds were made in a confluent monolayer by making a straight scratch in the center of the well using a sterile 200-µl pipette microtip. The scratched cells were removed by washing, and the experimental wounds created were treated with multiple concentrations of esmolol hydrochloride at 0.1, 1, 10, 100, and 500 µM and compared with the untreated and positive control (EGF 30 µM). The kinetics of migration of the cells in the wound was recorded by phase-contrast microscopy. The experiment was carried out in duplicate cells for each concentration.

### Animal study protocol

#### Efficacy evaluation of Galnobax for wound healing in streptozotocin-induced diabetic hairless rats

The effect of Galnobax^®^ in wound healing was evaluated in streptozotocin-induced diabetic hairless rats. Fifty male hairless rats were kept in standard autoclaved rodent cages with *ad libitum* food (Harland Tekland Irradiated Rodent Diet) and autoclaved water. The details of the animals used and selected for the study are provided in **Supplementary Table S1**. After acclimatization for 5 days, the rats were weighed, and initial glucose reading was taken using a blood glucose meter (Accu-Chek Aviva by Roche, code 991, test strip lot number: 301991, expiration date: 11/30/2010). Diabetes was induced in the rats by a single injection of streptozotocin at 35 mg/kg/day for 5 consecutive days. The diabetic state was confirmed after two consecutive high glucose readings of more than 350 mg/dl. Two sets of animals, each containing 21 animals, were taken for the study. The average weights for the two sets after randomization were 282.2 and 288.7 g, whereas the average glucose levels were 404.1 and 402.4 mg/dl, respectively. The rats were divided into five groups—namely, diabetic control, vehicle control (VC), and Galnobax at 7% (G7), 14% (G14), and 20% (G20). The wounds were created on the rat by using an Accupunch of 10 mm in diameter. Full-thickness skin was excised to get multiple wounds in the dorsal region (either six or four on each side of the spine). Galnobax, a topical gel formulation of esmolol hydrochloride (7, 14, and 20%) or vehicle was administered twice daily onto the wound.

The quality of healing was compared using laser Doppler to measure the blood flow in the wound on day 19. Laser Doppler flowmetry provides non-invasive, real-time measurements of the local tissue blood flow. The laser Doppler measurement uses the fact that, when laser light is reflected off a moving object such as a red blood cell, it undergoes a Doppler frequency shift, the amount of shift being dependent on the speed of the moving object. When laser light is used to illuminate the skin tissue, some of the light is scattered by the static tissue, and some of it is scattered by moving red blood cells. The total backscattered light contains a component that has not undergone any frequency shifting and a component that is frequency-shifted. These components mix together on the surface of a photodetector. The resulting photocurrent can then be processed to produce FLUX and CONC parameters related to the movement of the red blood cells. FLUX is related to the product of average speed and concentration of moving red blood cells in the tissue sample volume. The flux measurements were carried out using Moor Instruments MoorLAB laser Doppler monitor (Moor Instruments Inc., Wilmington, DE 19809, USA) on day 18 after wounding.

#### Biomarker assessment

In addition to wound healing, biomarkers like nitric oxide and hydroxyproline were measured from wound tissues to establish the mechanism of action of the drug. For this purpose, 42 rats were studied and divided into two groups. Set A had six wounds, two each in the three groups, *viz*., diabetic control, Galnobax 7%, and Galnobax 14%. Set B has four wounds, two each in the two groups, *viz*., diabetic control, vehicle control, and Galnobax 20%. Of the two wounds in each group, one was used for the estimation of nitric oxide and hydroxyproline content in the wound tissue. Seven animals per group were sacrificed on days 7, 14, and 19 for the estimation of hydroxyproline and nitric oxide contents.

#### Wound tissue homogenization

The tissue obtained from wounds were snap-frozen in liquid N_2_ and kept at -80°C in a freezer (the weight of the tissues was recorded). In total, 200 μl of cold phosphate-buffered saline (PBS) was added to the samples which weighed equal to and less than 20 mg, and 250 μl was added to the samples which weighed more than 20 mg. The samples were homogenized using a Dounce homogenizer, tissue fluid was passed through an Ultrafilter 10-kDa molecular weight cutoff filter at 14,000 × *g* at 4°C, and ultrafiltered tissue fluid was stored at -80°C until use (**Supplementary File**).

#### Measurement of endogenous nitrite and total nitrite

The assay measures the product of the enzymatic conversion of nitrate to nitrite by nitrate reductase, *viz*., nitric oxide. The detection of nitrite as an azo dye product of the Griess reaction was estimated by the colorimetric method. A seven-point standard curve was plotted using twofold serial dilutions in reaction diluent (1×). The standards were aliquoted for the standard curve with 50 μl/well reaction diluent (1×) used for blank, and 50 μl/standard point/well was used for the standard curve. The ultrafiltered sample was aliquoted, and 50 μl was added to each well. Moreover, 50 μl/well of reaction diluent (1×), 50 μl/well of Griess Reagent I, and 50 μl/well of Griess Reagent II were added to all wells and incubated at room temperature (RT) for 10 min. The optical density (OD) at 560 nm was read using DynexOpsys MR plate reader. For the total nitrite estimation, 50 μl ultra-filtered sample, 25 μl/well of NADH, and 25 μl/well of diluted nitrate reductase were added to the wells. The samples were mixed, and the wells were covered with an adhesive strip and incubated at 37°C for 30min. Subsequently, 50 μl/well of Griess Reagent I and 50 μl of Griess Reagent II were added to all wells, and the mixture was incubated at RT for 10 min. The OD at 560 nm was read using the DynexOpsys MR plate reader.

#### Hydroxyproline estimation from wound tissues

The hydroxyproline content from wound tissues is an indirect measurement of collagen turnover as well as the quality of collagen in the wound. Hydroxyproline from wound tissue was measured by the colorimetric method. Wound samples were weighed and homogenized with 5 μl/mg tissue of 0.5 ml 0.5 N acetic acid. We added 3× volume of 6 N HCl to each sample and hydrolyzed it at 105°C for 18 h. Furthermore, 50 μl of the hydrolyzed sample was neutralized with 24.25 μl of 10 N NaOH and diluted with177.75 μl 4× PBS and filtered through a 0.2-um filter to get a clear solution. Moreover, 50 μl of sample or hydroxyproline standards was taken in 1.5-ml tubes. Then, 450 μl of chloramine-T was added to each tube and incubated at RT for 25 min. In addition, 500 μl of p-dimethylaminobenzaldehyde solution was added to each tube and incubated at 65°C for 20 min. The samples were allowed to cool to RT for 10 min, and 200 μl was transferred to a 96-well ELISA plate. OD was measured with a spectrophotometer at 550 nm.

### Pharmacokinetics and toxicity study in miniature swine

#### Safety study of Galnobax gel in miniature swine with a 2-week recovery period

A total of 40 Hanford miniature swine (20 male and 20 female) divided into four treatment groups were used for this study. A 12-week topical application of Galnobax 20% was carried out in the miniature swine wound healing model with twice-daily dosing. Six wounds (10 cm^2^ each) of partial thickness were created on the dorsal region of all animals. Four wounds of each animal received twice-daily topical doses of either the vehicle or Galnobax gel, and the remaining two sites served as untreated comparative control wounds. Each group has four male and female miniature swine for the assessments and one each for the recovery period as given in **Table 1**. Safety parameters in terms of ECG, clinical pathology, wound site evaluation, and toxicokinetics were performed during the study.

**Table 1:** Study design for long-term (12 weeks) toxicity in miniature swine.

Test article(s)	Concentration	Dose		Toxicokinetic time points^a^
		Topical (mg/animal/dose)	SQ (mg/kg/dose)	
Vehicle Gel	NA	400 BID	NA	NA
Galnobax^®^	200 mg/g	400 BID	NA	Day 1, week 5/6: 0 (pre-dose), 15 30 60, 120, 240, and 480 min post-dose Week 12: 0 (pre-dose), 15, 30, 60, 120, 240, 480, 840, and 1,440 min post-final dose (morning)
Galnobax^®^ and esmolol (SQ)	200 mg/g topical and SQ dose—200 mg/ml	400 BID	30 once a day
		400 BID	30 BID

Each group contains four plus one male and female miniature swine animals.

a Blood was drawn before the second dosing of the day (at 8 h).

NA, Not Applicable.

#### Assessment of skin irritation and sensitization parameters

As Galnobax is a topical gel, tests were carried out to check its irritability on rabbit skin and its sensitization potential in the Guinea pig model for Galnobax 20% gel according to ISO 10993-10:2002. Three female New Zealand white rabbits were used for the study. Galnobax 20% was applied directly to the skin on each side of each rabbit. The negative control (physiological saline at 100%) and the positive control (2.5% sodium lauryl sulfate in saline) were each applied on one site of each animal. The sites were then covered by gauze sponges and a Kendall Webril^®^ pad and wrapped with 3-in. 3M Micropore™ tape to keep the test sites semi-occlusive. Each test site was scored individually after unwrapping at 60 min and at 24, 48, and 72 h for erythema and edema using the Draize skin scoring scale.

To assess the sensitization potential of Galnobax^®^ 20%, the Guinea Pig Closed Patch Sensitization Test (ISO) was performed. In total, 10 (five male and five female) and five (two male and three female) Hartley-strain guinea pigs were utilized in the test group and the control group, respectively. The animal received treatment topically for 6 h on the screening day and once per week subsequently for three consecutive weeks. At 2 weeks after the last topical application, the challenge dose application was made. For the challenge, Galnobax^®^ was applied at the highest non-irritating concentration which was 50% dilution of the test article. At the challenge, the test and control articles were kept at the challenge sites for 6 h. Observations of erythema, edema, and other effects were recorded at 24 and 48 h after the challenge applications.

### Statistical analysis

The data are presented in mean ± SD unless specified. One-way ANOVA was used for comparing the nitrite/nitrate oxide or hydroxyproline levels in the wounds, and Bonferroni’s *post-hoc* test was used for pairwise comparisons.

## Results

### Docking study

Esmolol was docked in the same cavity as that occupied by the co-crystallized ligand in 1EL3. The best scored docking pose from GRIP docking confirmed esmolol binding to AR mainly by hydrogen bonds with TYR48, HIS110, and pi- stacking interactions with PHE122 and TRP20. The docking score as well as ligand efficacy of esmolol was lower (-65.37 and -3.11) than the co-crystallized ligand (-63.24 and -2.34). This docking data showed that esmolol has the potential to bind to aldose reductase (docking pose shown in **Supplementary Figure S1**).

### *Ex vivo* experiments

**Figure 1:**
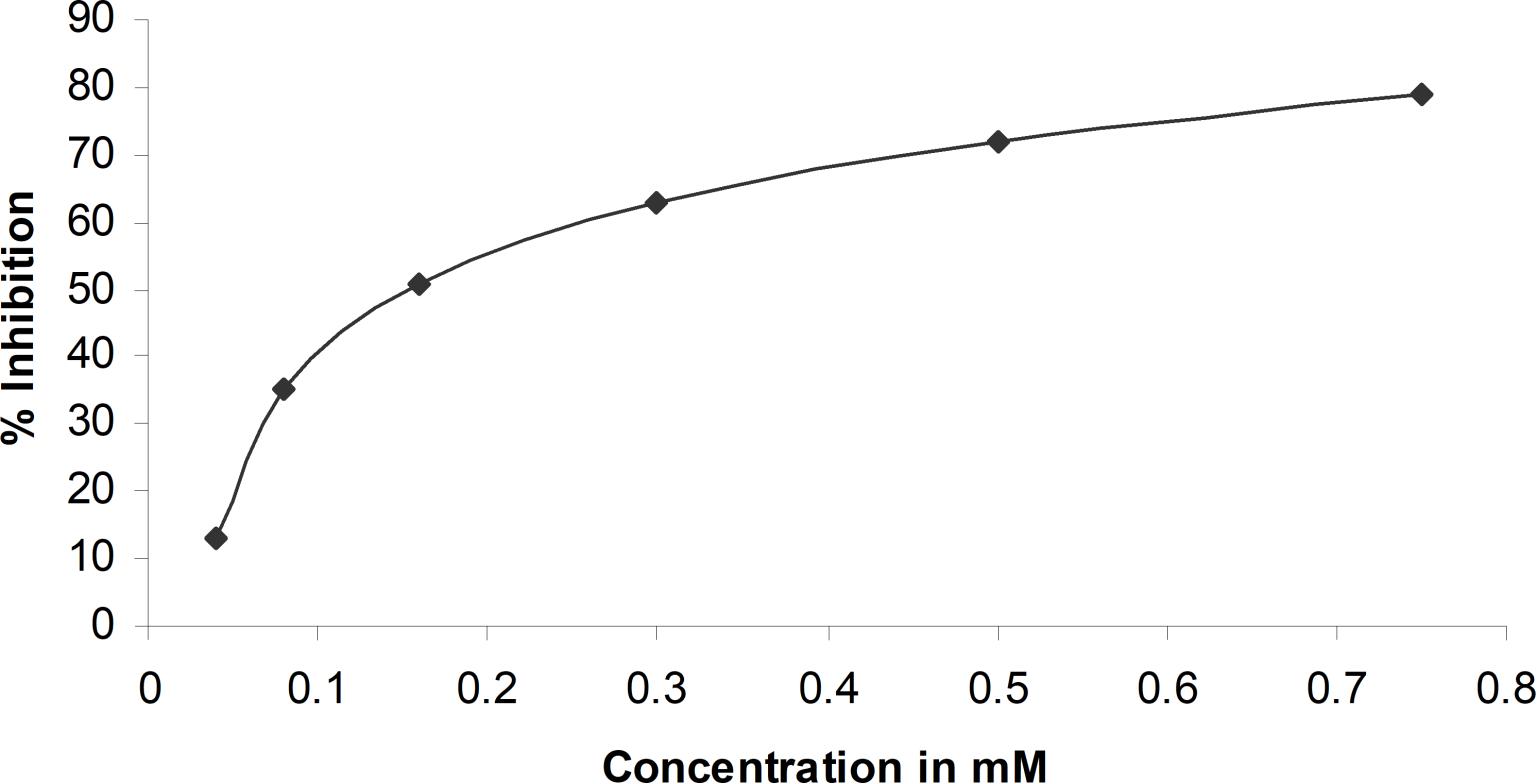
Percent inhibition of aldose reductase at different concentrations of esmolol hydrochloride.

For AR inhibitory activity, the percent of inhibition with esmolol hydrochloride was calculated considering the AR activity in the absence of the drug as 100%. The percent inhibition of aldose reductase measured at various concentrations of esmolol hydrochloride is reported in **Figure 1**. The IC_50_ of esmolol hydrochloride was estimated to be 150 µM. Since sorbitol formation is the direct product of the activity of aldose reductase, the inhibition of sorbitol formation in erythrocyte was estimated at IC50 concentration. Esmolol hydrochloride inhibits sorbitol formation in red blood cells by 59% when compared with glucose-induced sorbitol levels with respect to the sorbitol level at normal conditions (**Supplementary Figure S2**).

The formation of AGEs was effectively inhibited at a concentration of 1 mM esmolol hydrochloride as measured by the glycation of bovine serum albumin. The anti-glycation effect of esmolol was similar to that induced by the identical concentration of diclofenac, a known anti-glycation agent (**Table 2**), suggesting that esmolol hydrochloride is an inhibitor of AGE formation.

**Table 2:** Fluorescence measurements in the presence of esmolol hydrochloride.

Readings	Control	Diclofenac (1 mM)	Esmolol hydrochloride (500 µM)	Esmolol hydrochloride (1 mM)
1	7	2	6	4
2	7	0	9	0
3	7	3	6	3
4	8	3	2	4
5	8	3	2	2
Mean	7.5	2.5	5	2.6
SD	0.5	1.3	3.0	1.7

### *In vivo* results

**Figure 2:**
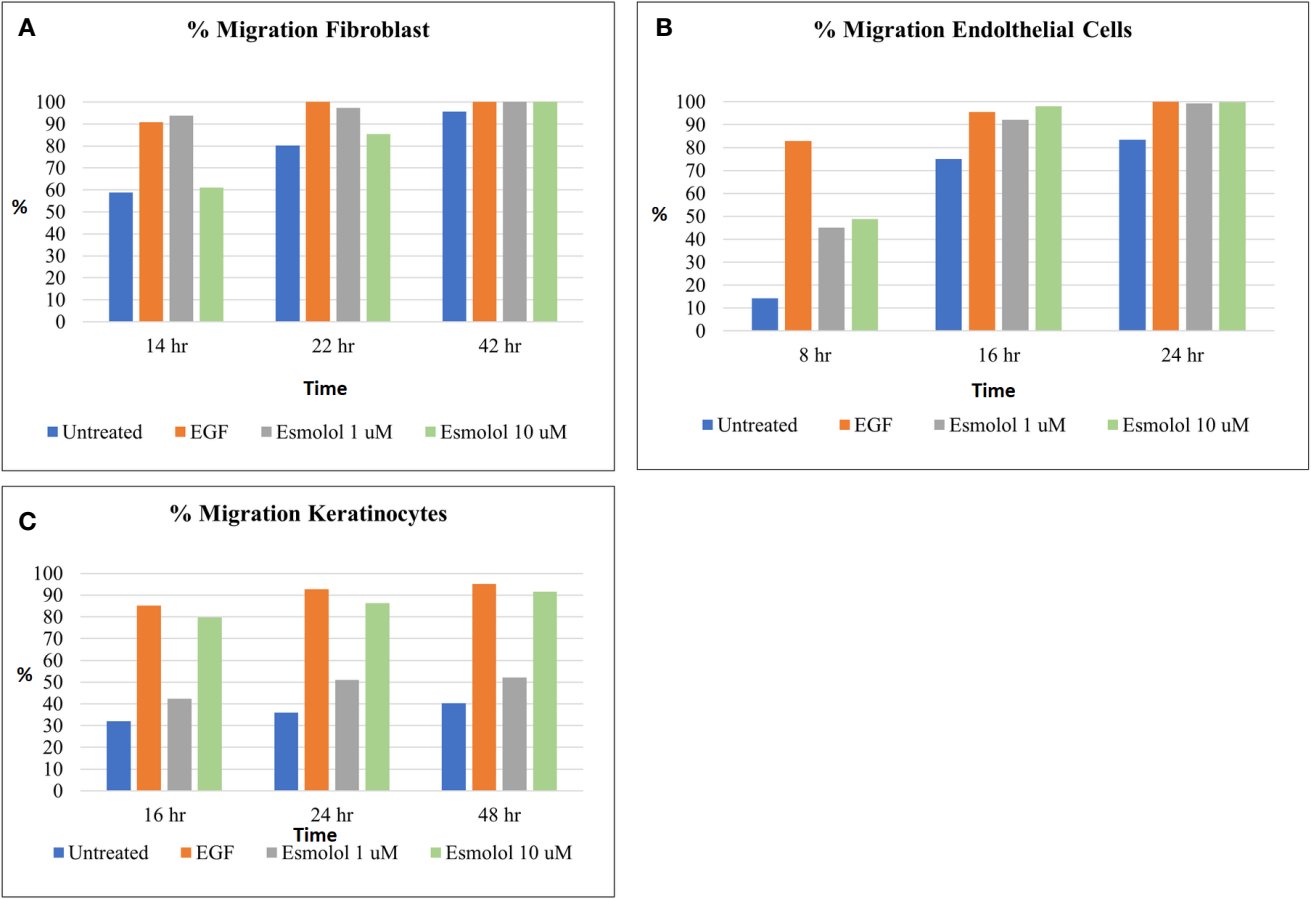
**(A–C)** Percentage migration of fibroblasts **(A)**, endothelial cells **(B)**, and keratinocytes **(C)** by different concentrations of esmolol hydrochloride (1 and 10 µM) and EGF (30 ng/ml, positive control).

The migration of human fibroblasts, endothelial cells, and keratinocytes (HFF-1, Ea.hy926, and HaCaT) at varied concentrations of esmolol hydrochloride and glucose is shown in **Figures 2A–C**. A lower migration of respective cells was observed at high concentrations of esmolol hydrochloride of 100 and 500 µM compared with the low concentrations of 1 and 10 µM. Esmolol hydrochloride at varied concentrations was also found to be non-cytotoxic at all the tested concentrations in all the cell lines.

### Animal study results

**Figure 3:**
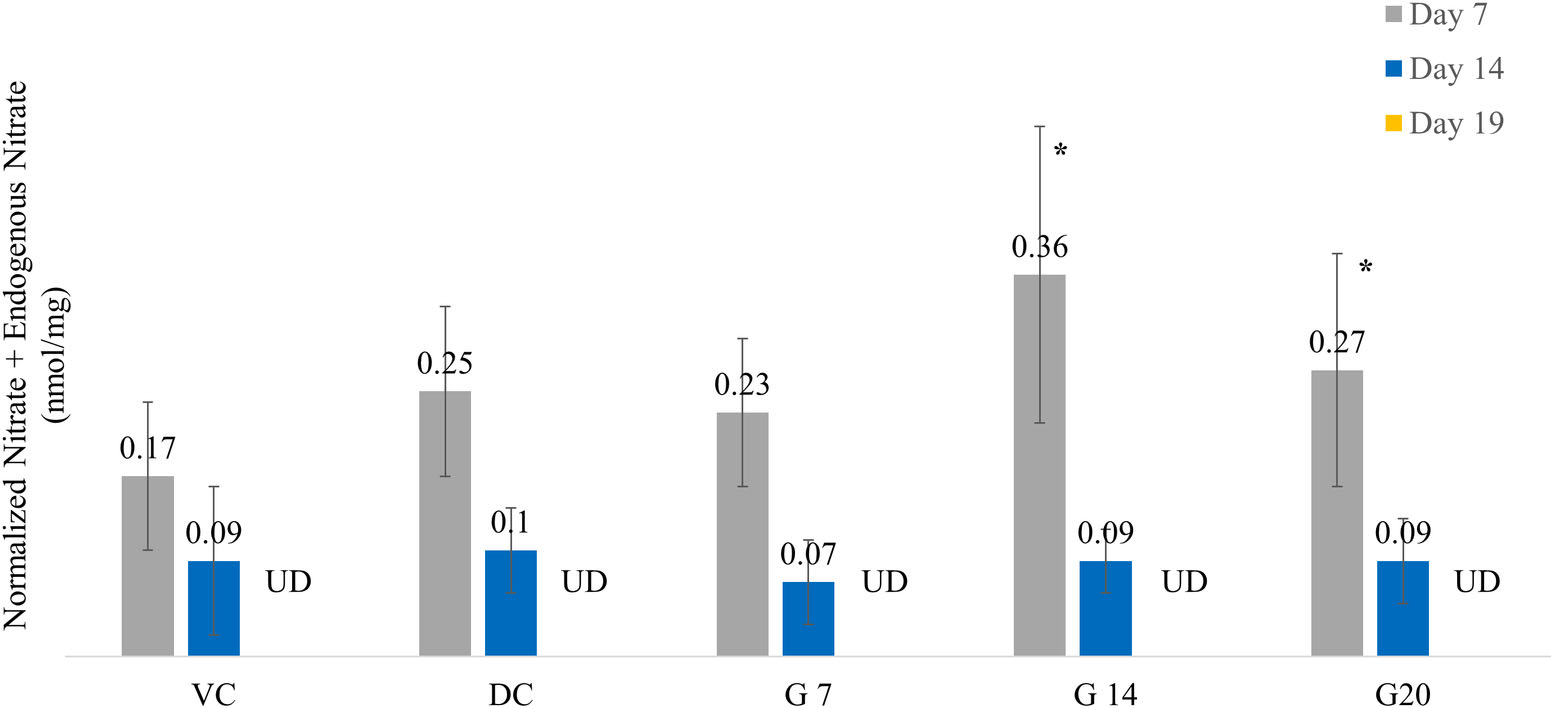
Nitric oxide levels across different treatment groups on days 7, 14, and 19. Asterisk indicates *p <*0.05 compared with the vehicle control group.

The nitric oxide (NO) levels measured on day 7 by total nitrite content in wound tissues of G14 and G20 were higher (*p* < 0.05) than those in the VC group. The G14 nitric oxide levels were 44 and 112% higher than the diabetic control and vehicle control, respectively. The G20 nitric oxide levels were 8 and 59% higher than those of the diabetic control and vehicle control, respectively. The average values of NO in the wound tissues of various groups are as shown in **Figure 3**.

**Figure 4:**
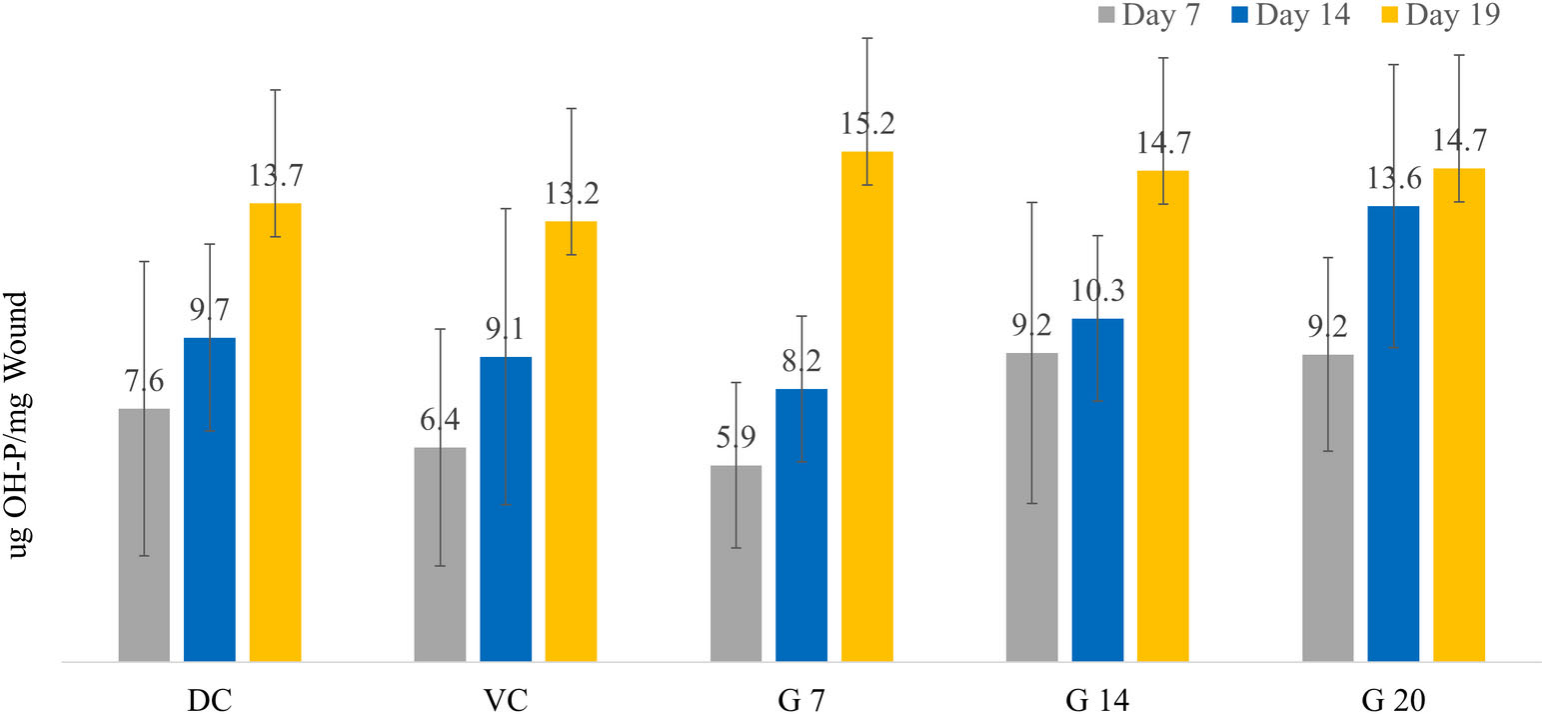
Shown are the hydroxyproline levels (μg/mg wound tissue) across different treatment groups on days 7, 14, and 19.

The average hydroxyproline measured from the wound tissues of various animal groups is shown in **Figure 4**. The hydroxyproline level measured on day 7 showed that the G14 and G20 groups have a higher collagen content compared with the control groups, though not statistically significant (*p* = 0.081). The day 7 hydroxyproline level in the wound was higher by 22 and 44% in G14 and by 21 and 43% in G20 compared with the diabetic control and vehicle control groups, respectively. The hydroxyproline level measured on day 14 was higher than that on day 7 in all groups. These levels were higher by 6 and 13% in G14 and by 41 and 49% in G20 compared with the diabetic and vehicle control groups (*p* = 0.062), respectively. Similar higher levels of hydroxyproline were observed on day 19 for all Galnobax groups (G7, G14, and G20) compared with the control groups.

The flux of blood flow for different groups measured on the epithelialized wound on day 19 after treatment is shown in **Table 3**. Intact non-diabetic skin exhibits the lowest flux, implying good vasculature as well as normal blood flow. The intact skins of diabetic animals also show a higher flux, indicating a disturbance in the vasculature of the animal. The average flux of the different groups shows that Galnobax 20% has improved healing compared with all other groups and is closer to normal intact diabetic skin (wound on day 19 was not completely closed). On the other hand, the vehicle control group exhibited poor healing since the flux is much higher in that group. The flux data clearly shows dose-dependent blood flow as well as vasculature improvement in the Galnobax treatment groups.

**Table 3:** Average flux of blood flow on the epithelialized skin in various groups.

Treatment	Mean flux (AU) ± SD
Intact non-diabetic skin	51.3 ± 93.8
Intact diabetic skin	77.7 ± 112.8
Diabetic control	122.5 ± 176.2
Vehicle control	186.5 ± 213.8
Galnobax, 7%	142.6 ± 155.0
Galnobax, 14%	122.5 ± 170.7
Galnobax, 20%	90.5 ± 124.6

### Animal wound closure data

**Figure 5:**
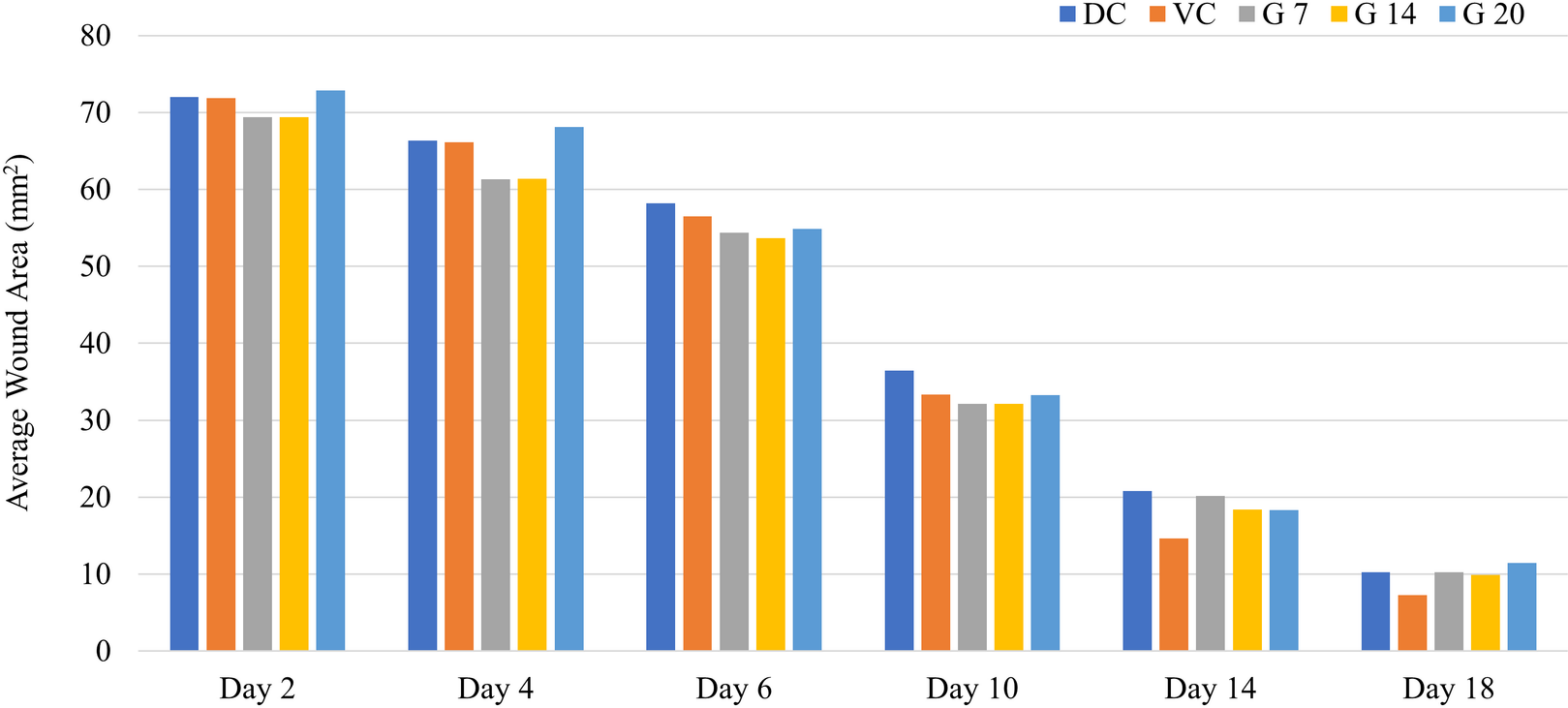
Day-wise wound area changes in the different treatment groups.

The percentage of epithelialization in the G7 and G14 groups was higher than in the control groups up to day 10 (**Figure 5**). However, after day 10, the vehicle control group shows enhanced epithelialization than the treatment groups. At the end of day 18, the vehicle control and G14 treatment groups show a similar wound closure, whereas the G7 and G20 groups show epithelialization similar to the diabetic control group.

### Toxicity and toxicokinetics studies of Galnobax

The 12-week dermal toxicity study in miniature swine showed that there were no abnormal local findings at the untreated or topically treated wound dose sites. Local findings at the SQ injection site areas were common but generally without complications. There was no indication that esmolol hydrochloride treatment interfered adversely with wound healing over the course of the study. Additionally, observation and modified Draize scoring were performed regarding the areas surrounding the wound dose sites. No erythema nor edema was observed. The mean *C*_max_ for a topical dose of Galnobax was 30 ng/ml, which was 30 times lower than that of the approved human dose (868–1,470 ng/ml) (21–23). After topical and subcutaneous administration, esmolol is rapidly converted to esmolol acid. However, the exposure to the parent drug, esmolol, was much lower than that of its metabolite, esmolol acid. There was no apparent accumulation of esmolol or esmolol acid after 12 weeks of twice-daily topical application, and esmolol was rapidly cleared from swine blood as shown in **Supplementary Table S3**. Additional results of dermal toxicity are provided in the **Supplementary File**.

There were no apparent gender differences in esmolol or esmolol acid toxicokinetic parameters and systemic exposure. However, in groups receiving topical and subcutaneous doses (30 and 60 mg/kg), female individuals showed a dose-dependent change in ovary weight without any histological changes in the ovaries. alnobax elicited a slight dermal response in the rabbit model under the conditioThus, the dermal toxicity study of Galnobax demonstrated its safety for long-term use (exposure of 12 weeks).

Galnobax elicited a slight dermal response in the rabbit model under the conditions of this test (the primary irritation index for Galnobax was 0.73). Galnobax did not elicit a skin sensitization reaction in the guinea pig model (the sensitization incidence and severity were 0.0 for Galnobax 20% gel at 24 and 48 h).

## Discussion

The present study clearly indicates the pleotropic mechanism of action of esmolol hydrochloride (Galnobax) for the healing of wounds in a diabetic condition. The novel action of esmolol hydrochloride showed that it inhibits aldose reductase and the formation of sorbitol as well as AGEs. The ability of esmolol hydrochloride to promote the migration of human fibroblasts, endothelial cells, and keratinocytes under a high-glucose environment showed its promise in the healing of diabetic wounds.

The polyol pathway is involved in the mechanism of various organ injuries induced by a high concentration of glucose. Hyperglycemia enhances the polyol pathway, resulting in the accumulation of sorbitol in the cells that causes organ injury by high osmotic pressure and oxidative stress. Higher levels of sorbitol further promote the formation of AGEs, the release of proinflammatory cytokines, an increase in reactive oxygen species, and cell damage (10) causing diabetic neuropathy. The present study demonstrates that esmolol effectively inhibits aldose reductase and the formation of sorbitol, which may improve the local wound environment. Furthermore, it has been established that AGEs induce autophagy in macrophages, and modulation in macrophage polarization negatively impacts cutaneous wound healing (24). The present study demonstrates that esmolol effectively inhibits the formation of AGEs and thereby shall inhibit autophagy in macrophages and reduce cellular oxidative stress, leading to improvements in diabetic wound healing.

NO induction triggers collagen production as well as promotes cell proliferation and angiogenesis, thereby helping the closure of wounds (25). The concentration of NO is significantly reduced in chronic diabetic ulcers. The animal experiment in the present study clearly showed higher NO induction (*p* < 0.05) and collagen turnover by the application of Galnobax. The enhanced endogenous nitric oxide promotes endothelial progenitor cell (EPC) migration from the bone marrow and homing to the wound site. This leads to an increase in fibroblasts and macrophages into the wound bed. The EPC homing promotes endothelial cell migration, leading to neovasculogenesis and wound healing (25). Furthermore, endogenous nitric oxide can act as a vasodilator that can help carry blood as well as nutrients to the wound site. The induction of angiogenesis or local vasculature has been evaluated in the present study using laser Doppler measurement of blood flow showing a better vasculature by Galnobax treatment over vehicle control. The nitric oxide level peak was observed on day 7 for the G14 group, which may aid chronic wounds that are generally stuck in the inflammatory phase as well as help in the stimulation of proliferative processes responsible for the healing of wounds. However, on day 14, the NO levels in all the groups were similar, while on day 19, none of the groups showed detectable levels of NO. Thus, Galnobax induces nitric oxide at the appropriate stage of wound healing followed by a decline in nitric oxide expression when it may not be required. It should be noted that nitric oxide patches have not demonstrated a significantly complete wound closure in clinical trials for DFU since they continue to provide NO in the proliferative as well as remodeling phases of wound healing, which may be detrimental for achieving the complete closure of wounds (26).

Hydroxyproline steadily increases in all groups as wound healing progresses; however, the treatment groups show a higher collagen content on all days of measurement. In the remodeling phase of wound healing on day 14, the treatment groups show a dose-dependent enhancement in collagen content in the wound. This could be highly crucial since non-healing wounds have delayed hydroxyproline (collagen) production. The delayed collagen synthesis is also seen in the control groups, as they do not significantly produce collagen at the wound site. The higher hydroxyproline in the wound area of treatment groups implies a higher collagen content and having good stability, which would help in better remodeling and healing of wounds.

In addition to the above-mentioned observed effects of Galnobax, several other known actions of esmolol promise its use in chronic wound healing such as DFU. It is known that esmolol hydrochloride inhibits caspase-3 and upregulates Bcl-2, thereby preventing necrosis in the wound bed (27). A study on healthy, diabetic, and DFU subjects has shown that the expression of proapoptotic markers caspase-3 and Bax was higher, and the expression of anti-apoptotic marker Bcl-2 was lower in the DFU participants, leading to necrosis of wound tissue (28). Esmolol helps in the movement of epithelial cells during diabetic wound closure through the activation of ERK1/2 (29). Esmolol upregulates neurokinin receptor and releases substance P; this may help in vasodilation and improves local blood supply (30, 31), thereby helping DFU healing. Esmolol hydrochloride has demonstrated its ability to attenuate cytokine (IL-6 and TNF-α) levels as well as CRP after laparoscopic gastrectomy (32, 33). A plasma cytokine analysis of DFU patients showed higher median plasma levels in DFU patients (34, 35) for interleukin-6 (IL-6), high-sensitivity C-reactive protein, and tumor necrosis factor-alpha (TNF-α), thus impeding wound healing. Esmolol was reported to lower the tissue accumulation of lactate by exogenous lactate clearance (36). The diabetic wound healing has shown lactate concentrations of ~10–12 mM as opposed to 1–3 mM normally found in blood and most uninjured resting tissues (37, 38). Esmolol showed antioxidant properties by the reduction of malondialdehyde and glutathione peroxidase levels in a clinical study (39).

This data shows that there is no significant advantage in wound closure in the treatment groups over the control groups. Therefore, the enhanced expression of markers of wound healing (nitric oxide, hydroxyl-proline, and laser Doppler flux) due to Galnobax treatment should be considered to quantify wound healing. Based on existing information about esmolol hydrochloride and the preclinical experiments conducted using Galnobax, it is evident that 14 and 20% Galnobax would be a promising treatment option for diabetic foot ulcer(s).

Esmolol hydrochloride is an approved intravenous drug, and considerable experience with its use is present. However, the toxicity information required to establish the safety of esmolol *via* topical route was established in the present study. The non-clinical toxicity and pharmacokinetic studies demonstrated that Galnobax is safe for long-term dermal use in the clinic and shows low systemic absorption. As esmolol is minimally absorbed into the systemic circulation (upon topical application of Galnobax) and is retained in higher amounts in the wound site, esmolol can induce and upregulate several essential wound healing processes such as nitric oxide and collagen production, which led to the therapeutic effect observed in this study.

Overall, the efficacy of Galnobax is defined in terms of the induction of internal wound healing processes by a higher endogenous NO induction at the wound site in the inflammatory phase, improved collagen turnover in the proliferative and remodeling phase, neo-vascularization induction supported by better vasculature, and induction of cellular migration, thereby resulting in wound matrix development and remodeling. Thus, based on existing information about esmolol hydrochloride and the preclinical experiments conducted using Galnobax, it is evident that Galnobax would be a promising treatment option for diabetic foot ulcers, when it is known that DFU are associated with considerable morbidity and mortality (40).

In conclusion, esmolol hydrochloride (Galnobax) treatment having a novel pleotropic mechanism would be a new option for the clinicians to treat diabetic foot ulcer. Galnobax 14% and Galnobax 20% along with the standard of care need to be investigated in a clinical study for its efficacy and safety.

## Data availability statement

The original contributions presented in the study are included in the article/**Supplementary Material**. Further inquiries can be directed to the corresponding author.

## Ethics statement

The animal study was reviewed and approved by the animal research institute committee (IACUC of Bio-Quant Inc.).

## Author contributions

AR, SK, and SD were involved in the design of the study and various experimental protocols. SK and SD performed the animal experiments. AR and SK wrote the draft of the manuscript. AR shall be the guarantor of the manuscript. All authors contributed to the article and approved the submitted version.

## Funding

This work received funding from NovaLead Pharma Pvt. Ltd. and Biotechnology Industry Research Assistance Council, New Delhi, set up by the Department of Biotechnology, Government of India.

## Acknowledgments

The authors acknowledge support from NovaLead team members at various stages.

## Conflict of interest

Authors SK and SD were employed by NovaLead Pharma Pvt. Ltd., Pune, India.

The authors declare that this study received funding from NovaLead Pharma Pvt. Ltd. The funder was not involved in the study design, collection, analysis, interpretation of data, the writing of this article or the decision to submit it for publication.

## Publisher’s note

All claims expressed in this article are solely those of the authors and do not necessarily represent those of their affiliated organizations, or those of the publisher, the editors and the reviewers. Any product that may be evaluated in this article, or claim that may be made by its manufacturer, is not guaranteed or endorsed by the publisher.
