# Supplementary material for: Novel topical esmolol hydrochloride improves wound healing in diabetes by inhibiting aldose reductase, generation of advanced glycation end products, and facilitating the migration of fibroblasts
Source: Front Endocrinol (Lausanne). 2022 Aug 23;13:926129. doi: 10.3389/fendo.2022.926129 (PMC9446078; doi:10.3389/fendo.2022.926129)
Supplement: Supplementary file 1 [file Table_1.docx]

**Supplementary Information**

**Cytotoxicity Protocol in cell cultures: Determination of non-cytotoxic concentrations of Esmolol hydrochloride**

1. HaCaT, HFF-1 and Ea.hy926 cells were counted and plated in 96 well plates at the density corresponding to 5 X 10^3^ cells/well/180 μl of cell growth medium.

2. The above cells were then incubated overnight under growth conditions of 37°C, 95 % Humidity, 5 % CO2.so as to allow the cell recovery and exponential growth.

3. Following overnight incubation, the above cells were serum starved in DMEM (containing 25 mM glucose) supplemented with 10 % FBS for 24 h.

4. Following serum starvation, the cells were treated with Esmolol hydrochloride at various concentrations (0.01 to 1000 μM).

5. The Ea.hy926 cells were then incubated for 24 h and HaCaT and HFF-1 cells were incubated for 48 h in CO2 incubator at 37 °C, 5 % CO2 and 95 % humidity.

6. Following incubation, the plates were taken out and 20 μl of 5 mg/ml of MTT 3-(4,5-dimethythiazol-2-yl)-2,5-diphenyl tetrazolium bromide solution was added to all the wells followed by additional incubation for 3 h at 37 °C. The supernatant was aspirated and 150 μl of DMSO was added to each well to dissolve formazan crystals. The absorbance of each well was then read at 540 nm using Synergy HT micro plate reader.

7. The percentage cytotoxicity at each tested concentration was calculated using the following formula:

% Cytotoxicity = (1-X/R)*100

Where X = Absorbance of treated cells

R = Absorbance of untreated cells

8. The concentrations exhibiting % cytotoxicity of < 30 % was considered as non-cytotoxic.

The percentage cellular cytotoxicity obtained in HaCaT, HFF-1 and Ea.hy926 cells treated with different concentrations of Esmolol hydrochloride (0.01 to 1000 μM) is provided in Table below.

| **Test Concentration of Esmolol** | **% Cytotoxicity w.r.t. untreated cells** | | |
| --- | --- | --- | --- |
|  | HaCaT | HFF-1 | Ea.hy926 |
| 0.01 | -19.20 | -21.56 | 5.84 |
| 0.1 | -8.02 | -10.67 | 1.73 |
| 1 | -6.93 | -8.29 | 11.37 |
| 10 | -14.81 | -17.06 | 13.76 |
| 50 | -18.11 | -21.77 | 3.85 |
| 100 | -18.52 | -21.91 | 2.79 |
| 500 | 16.19 | 14.04 | 17.48 |
| 1000 | 18.31 | 16.57 | 12.74 |

**S1Protocol: Wound healing and closure in diabetic rats**

Fifty male hairless rats were kept in standard autoclaved rodent cages with *ad libitum* food

(Harland Tekland Irradiated Rodent Diet) and autoclaved water. The details of animals used and selected for the study are provided in **Table S1** below. After acclimatization for 5 days, rats were weighed and initial glucose reading was taken using blood glucose meter (Accu-Chek Aviva by Roche, Code 991, Test Strip: Lot number: 301991, Expiration Date: 11/30/2010). Diabetes was induced in the rats by a single injection of Streptozotocin (STZ) 35mg/kg/day for 5 consecutive days. The diabetic state was confirmed after 2 consecutive high glucose readings of more than 350 mg/dL. Two sets of animals, each containing 24 animals were taken for the study. The average weights for two sets after randomization were 282.2 and 288.7 g, whereas average glucose levels were 404.1 and 402.4 mg/dL, respectively. The wounds were created on the rat by using an Accupunch of 10 mm diameter. Full-thickness skin was excised to get multiple wounds in the dorsal region (either six or four on each side of the spine). The experiment also included diabetic controls and vehicle control groups along with the treatment groups. The study was terminated on day 19 after wounding.

**Dosing:** Galnobax^®^, a topical gel formulation of Esmolol hydrochloride (7%, 14% and 20%)

was administered twice daily onto the wound. Vehicle gel was also applied twice daily on to the wound.

**Table S1: Test System of the Type II Diabetic Hairless Male Rats**

| Species/Strain | Hairless Rats |
| --- | --- |
| Physiological state | Diabetic |
| Entrance into study | Stable Streptozotocin-Induced Type II diabetic rats with 2  consecutive high glucose readings were required for  selecting the animals to enter the study |
| Weight range at start of study | Average of 293 g |
| Sex | Male |
| Animal supplier | Harlan, USA |
| Number of animals/group | 21 |
| Randomization | Into five groups as described below |
| Replacement | No animals were replaced during the course of the study |

The experimental grouping of a total of 42 animals was done as follows;

Group 1: Diabetic Control

Group 2: Vehicle Control

Group 3: Galnobax-7%

Group 4: Galnobax-14%

Group 5: Galnobax-20%

Two sets of animals were taken with 21 animals in each set. Set A has six wounds two each of three groups, viz., Diabetic Control, Galnobax-7%, Galnobax-14%. Set B has four wounds two each of two groups, viz., Diabetic Control, Vehicle Control, Galnobax-20%. Seven animals per group were sacrificed on days 7, 14 and 19 for estimation of endpoints such as hydroxyproline and nitric oxide contents. Of the two wounds in each group, one was used for estimation of nitric oxide and hydroxyproline content in the wound tissue. The other wound was utilized for histological evaluation. Several wound healing parameters were measured to evaluate the wound healing capability of the topical gel formulation.

**S2 Protocol: Procedure for measurement of biomarkers in wound healing**

**1. Wound tissue homogenization**

a. Wound tissues were snap-frozen in liquid N2 and kept in -80ºC freezer

b. The weight of tissues was recorded

c. 200μl cold Phosphate-buffered saline (PBS) was added to samples which weighed equal to and less than 20mg and 250μlwas added to samples which weighed more than 20mg.

d. The samples were homogenized using a Dounce homogenizer.

e. Tissue fluid was passed through an Ultrafilter 10kDa molecular weight cut-off filter @

14,000xg, 4ºC.

f. Ultrafiltered tissue fluid was stored @ -80ºC until use.

**2. Measurement of endogenous Nitrite by R&D System Kit - Nitrite assay**

a. Preparation of nitrite standard curve: A seven point standard curve was plotted using 2-fold

serial dilutions in Reaction Diluent (1X)

b. The Standards were aliquoted for the Standard curve: 50μl/well Reaction Diluent (1X) was

used for blank, and 50μl/standard point/well was used for standard curve.

c. The ultrafiltered sample was aliquoted and 50μl was added to each well.

d. 50μl/well of Reaction Diluent (1X) was added to all wells.

e. 50μl/well of Griess Reagent I and 50μl/well of Griess Reagent II was added to all wells and

incubated at RT for 10min.

f. The OD 560nm was read using DynexOpsys MR plate reader.

**3. Measurement of Total Nitrite by R&D System Kit - Nitrate reduction assay**

a. Preparation of nitrate standard curve: A seven-point standard curve was prepared using 2-

fold serial dilutions in Reaction Diluent (1X)

b. The standards were aliquoted for the Standard curve and 50μl/well Reaction Diluent (1X) was used for blank and 50μl/standard point/well for standard curve.

c. The ultra-filtered sample was aliquoted and 50μl was added to each well.

d. 25μl/well of NADH was added to all wells.

e. 25μl/well of diluted Nitrate Reductase was added to all wells. The samples were mixed well

and the wells were covered with the adhesive strip and incubated at 37ºC for 30min

f. Add 50μl/well of Griess Reagent I and 50μl/well of Griess Reagent II to all wells; incubate

at RT for 10min.

g. The OD560nm was read using the DynexOpsys MR plate reader.

**4. Protocol for hydroxyproline estimation from wound tissues**

Hydroxyproline from wound tissue was measured by colorimetric method. This method is a

simple and reproducible method for hydroxyproline measurement.

1. Wound samples were weighed and homogenized with 5μl/mg tissue 0.5 ml 0.5N Acetic Acid.

2. Added 3X volume of 6N HCl to each sample and hydrolysed at 105ºC for 18h.

3. 50 μl of hydrolysed samples were neutralized with 24.25 μl 10N NaOH and diluted with

177.75 μl 4xPBSand filtered through a 0.2 um filter to get clear solution.

4. 50μl sample or Hydroxyproline standards were taken in 1.5ml tubes.

5. 450μl of Chloramine-T was added to each tube and incubated at RT for 25 minutes.

6. 500μl of p-dimethylaminobenzaldehyde solution was added to each tube and incubated at

65ºC for 20 minutes.

7. Samples were allowed to cool to RT for 10 minutes and 200μl were transferred to 96 well

ELISA plate.

9. OD was measured in a spectrophotometer at 550 nm.

**5. Protocol for laser Doppler flowmetry for blood flow measurement**

Laser Doppler flowmetry provides non-invasive, real-time measurements of local tissue bloodflow. The Laser Doppler measurement uses the fact that when laser light is reflected off amoving object such as a red blood cell it undergoes a Doppler frequency shift, the amount ofshift being dependent on the speed of the moving object.When laser light is used to illuminate the skin tissue some of the light is scattered by the statictissue, and some scattered by moving red blood cells. The total backscattered light contains acomponent that has not undergone any frequency shifting and a component that is frequencyshifted. These components mix together on the surface of a photodetector. The resultingphotocurrent can then be processed to produce FLUX and CONC parameters relating to themovement of the red blood cells.FLUX is related to the product of average speed and concentration of moving red blood cells inthe tissue sample volume. Flux is measured in arbitrary units.Flux measurements were carried out using laser Doppler of Moor Instruments moorLAB laserDoppler monitor (Moor Instruments Inc., Wilmington, DE 19809, USA) on day 18 afterwounding.

**S3 Protocol: Skin irritation of Galnobax^®^ in rabbit model**

3 female New Zealand White rabbits were used for the study. Galnobax-20% was applied directly to the skin on each side of each rabbit. The negative control (physiological saline at 100%) and the positive control (2.5 % sodium lauryl sulfate in saline) were applied, each on 1 site of each animal. The sites were then covered by gauze sponges and a Kendall Webril^®^ pad, wrapped with Three-inch 3M Micropore™ tape to keep the test sites semi-occlusive. Each test site was scored individually after unwrapping at 60 minutes, 24, 48 and 72 hrs, for erythema and edema using the Draize skin scoring scale given in table below.

**Draize scoring scale for skin reactions**

| Category | Score | Description |
| --- | --- | --- |
| Erythema formation | 0 | No erythema |
|  | 1 | Very slight erythema (barely perceptible) |
|  | 2 | Well-defined erythem |
|  | 3 | Moderate to severe erythema |
|  | 4 | Severe erythema (beet redness) to slight eschar formation (injuries in depth) |
|  | **Total erythema score = 4** | |
| Edema formation | 0 | No edema |
|  | 1 | Very slight edema (barely perceptible) |
|  | 2 | Well-defined edema (edges of area well-defined by definite raising) |
|  | 3 | Moderate edema (area raised approximately 1 mm) |
|  | 4 | Severe edema (area raised more than 1 mm and extending beyond area of exposure) |
|  | **Total edema score = 4** | |
| **Total possible primary irritation score = 8** | | |

The dermal irritation response categories in rabbit model are given below

| Response category | Response |
| --- | --- |
| Negligible | 0.0 to 0.4 |
| Slight | 0.5 to 1.9 |
| Moderate | 2.0 to 4.9 |
| Severe | 5.0 to 8.0 |

This test was carried out under Good Laboratory Practices (GLP) conditions.

**S4 Protocol: Skin sensitization of Galnobax^®^ in Guinea pig model**

To assess the sensitization potential of Galnobax^®^20%, the Guinea Pig Closed Patch Sensitization Test (ISO) was performed. 10 (5M, 5F) and 5 (2M, 3F) Hartley-strain guinea pigswere utilized in the test and control group, respectively. The animal received treatment topically for 6 hours on the screening day and once per week subsequently for three consecutive weeks. Two weeks after the last topical application, the challenge dose application was made. For the challenge, the Galnobax^®^ was applied at the highest non-irritating concentration which was 50% dilution of the test article. At the challenge, test and control articles were kept at the challenge sites for six (6) hours. Observations of erythema, edema and other effects were recorded 24 and 48 hours after the challenge applications using Draize scores.

This test was carried out under Good Laboratory Practices (GLP) conditions.

**Additional results of 12-week dermal toxicity study in miniature swine**

***Toxicokinetic of Topical Galnobax^®^***

After the first topical dose of Galnobax^®^ Gel 400 mg, Esmolol C_max_ was 30.3 ng/mL and t_max_ was 0.3 hr (18 min). Blood concentrations of Esmolol were below the quantitation limit (5 ng/mL) by the second daily dose (8 hr). There was no apparent accumulation of Esmolol after 12 weeks of twice daily topical application.

Esmolol is rapidly converted to Esmolol acid. After the first topical dose of Galnobax^®^ Gel 400 mg, Esmolol acid appeared in the blood at 15 min, reached the C_max_ of 683 ng/mL at 2.6 hr. Esmolol acid was detected at 8 hr post dose with the t_1/2_ of approximately 4 hr. The exposure of Esmolol acid (C_max_ and AUC_last_) was much lower after repeat application of Galnobax^®^ Gel.

**Table S2 - Mean Esmolol and Esmolol Acid Toxicokinetic Parameters in Group 2 Swine Receiving Topical 400 mg Galnobax^®^ Gel**

| Compound | Period |  | Cmax  (ng/mL) | Tmax  (hr) | t1/2  (hr) | AUClast  (hr*ng/mL) | AUC0-8  (hr*ng/mL) |
| --- | --- | --- | --- | --- | --- | --- | --- |
| Esmolol | Day 1 | Mean | 30.3 | 0.30 | - | 18.7 | - |
|  |  | sd | 17.3 | 0.11 | - | 11.3 | - |
|  | Week 6 | Mean | - | - | - | - | - |
|  |  | sd | - | - | - | - | - |
|  | Week 12 | Mean | 140 | 0.38 | - | 17.5 | 35.4 |
|  |  | sd | 190 | 0.18 | - | 23.8 | 47.1 |
| Esmolol  Acid | Day 1 | Mean | 683 | 2.60 | 4.18 | 3780 | - |
|  |  | sd | 284 | 0.97 | 0.75 | 1520 | - |
|  | Week 6 | Mean | 52.5 | 5.33 | - | 308 | - |
|  |  | sd | 25.2 | 2.07 | - | 184 | - |
|  | Week 12 | Mean | 59.7 | 2.09 | 15.90 | 291 | 269 |
|  |  | sd | 33.3 | 1.66 | 8.09 | 256 | 161 |

-: Insufficient data to determine.

The topical administration of Galnobax^®^ Gel (with dose active ingredient dose of 400 mg/dose, 800 mg/day) to surgically created wounds in miniature swine was not associated with effects related to body weight, food intake, abnormal clinical observations, impairment of wound healing, or biologically significant changes in clinical pathology or organ weight parameters when compared with administration of vehicle gel. The administration of a single or two daily SQ Esmolol dose 30 mg/kg greatly increased Esmolol and Esmolol acid systemic exposure.Significant findings in animals that received exposure‑potentiating doses of subcutaneously administered Esmolol included the nearly universal development of moderate to severe local reactions at the injection sites, as well as a reduction in absolute and relative ovary weights. The no observed effect level (NOEL) for this study was 800 mg/day for topical only application and the no observed adverse effect level (NOAEL) was greater than 800 mg/day for topical plus two 30 mg/day subcutaneous administrations, discounting the statistically significant reductions in ovary weights as there were no observed adverse histopathologic changes associated with this finding. Therefore, from the results of this study, topical exposure to Esmolol byadministering Galnobax^®^ gel on a significantly large enough wound site does not result in substantial systemic exposure to Esmolol nor does it result in any histopathologic changes.In contrast, the topical Galnobax^®^ dose and additional SC injection of Esmolol to the maximum amount feasible and maximum systemic exposure achievable (greater than 100-fold based on toxicokinetic results) essentially demonstrate the only potential worst-case effects if substantial exposure to Esmolol from Galnobax^®^ gel topical application could occur. However, for diabetic wound treatment by the topical dose of Galnobax^®^, such dose level that may lead to maximum exposure is obviously not achievable.Both Esmolol and Esmolol acid do not accumulate in any of the doses used in the study. Thus, the dermal toxicity study of Galnobax^®^ demonstrated its safety for long term use (exposure of 12-weeks).

**Modified Draize Scoring System**

| Category | Score | Description |
| --- | --- | --- |
| Erythema | 0 | No erythema |
|  | 1 | Slight erythema |
|  | 2 | Well-defined erythema |
|  | 3 | Moderate or severe erythema |
|  | 4 | Severe erythema or slight eschar formation (injuries in depth) |
| Edema | 0 | No edema |
|  | 1 | Very slight edema |
|  | 2 | Slight edema (well-defined edges) |
|  | 3 | Moderate edema (raised > 1 mm) |
|  | 4 | Severe edema (raised > 1 mm and extending beyond the area of exposure) |

**S1 Figure: Docked pose of Esmolol in Aldose Reductase (**(The drug is shown in stick model and hydrogen bonding interactions by yellow lines)

**S2 Figure:** Sorbitol concentration in erythrocytes amongst the groups
